# Towards bioprocess engineering of cable bacteria: Establishment of a synthetic sediment

**DOI:** 10.1002/mbo3.1412

**Published:** 2024-05-06

**Authors:** Judith Stiefelmaier, Joshua Keller, Wiebke Neupert, Roland Ulber

**Affiliations:** ^1^ Chair of Bioprocess Engineering, RPTU Kaiserslautern‐Landau Kaiserslautern Germany

**Keywords:** cable bacteria, *Candidatus* Electronema aureum GS, electrochemical bioprocess engineering, synthetic sediment

## Abstract

Cable bacteria, characterized by their multicellular filamentous growth, are prevalent in both freshwater and marine sediments. They possess the unique ability to transport electrons over distances of centimeters. Coupled with their capacity to fix CO_2_ and their record‐breaking conductivity for biological materials, these bacteria present promising prospects for bioprocess engineering, including potential electrochemical applications. However, the cultivation of cable bacteria has been limited to their natural sediment, constraining their utility in production processes. To address this, our study designs synthetic sediment, drawing on ion exchange chromatography data from natural sediments and existing literature on the requirements of cable bacteria. We examined the effects of varying bentonite concentrations on water retention and the impacts of different sands. For the first time, we cultivated cable bacteria on synthetic sediment, specifically the freshwater strain *Electronema aureum* GS. This cultivation was conducted over 10 weeks in a specially developed sediment bioreactor, resulting in an increased density of cable bacteria in the sediment and growth up to a depth of 5 cm. The creation of this synthetic sediment paves the way for the reproducible cultivation of cable bacteria. It also opens up possibilities for future process scale‐up using readily available components. This advancement holds significant implications for the broader field of bioprocess engineering.

## Graphical abstract

In this study, we successfully cultivated the cable bacteria strain *Electronema aureum* GS on a newly developed synthetic sediment, marking the first instance of such cultivation on synthetic rather than natural sediment. The combination of this synthetic sediment and a specially designed sediment bioreactor enables reproducible cultivation of cable bacteria. This advancement paves the way for future investigations into the potential application of cable bacteria in bioprocess engineering.

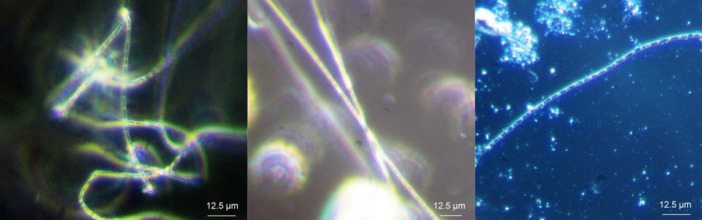

## 1 INTRODUCTION

### 1.1 Cable bacteria as promising microorganisms for bioprocess engineering

Cable bacteria are multicellular microorganisms that can transport electrons over distances of several centimeters. The filamentous bacteria have only been known for about 12 years (Pfeffer et al., 2012), and have been found in the sediment of numerous freshwaters and marine sediment (Burdorf et al., 2017; Risgaard‐Petersen et al., 2015). They grow up to several centimeters deep into the sediment (Pfeffer et al., 2012; Schauer et al., 2014), whereby the cells oxidize sulfur compounds in the deeper, anoxic sediment. The released electrons are conducted along the filament into the oxic sediment layer, where the cells reduce oxygen (Bjerg et al., 2018). By transporting electrons from cell to cell via the shared periplasmic space, cable bacteria can use electron donors and acceptors that are spatially separated from each other. This offers cable bacteria an advantage for survival in aquatic sediments (Meysman, 2018). The individual cells are connected by a shared periplasm in which conductive fibers run along the entire filament. This enables the transfer of released electrons over a distance of several centimeters (Thiruvallur Eachambadi et al., 2020). In doing so, the filament sheath exhibits the highest conductivity for biological materials described to date (Meysman et al., 2019). The unique metabolism of cable bacteria, including the ability for CO_2_ fixation (Kjeldsen et al., 2019), and their ability to conduct electrons over long distances makes them interesting production organisms for bioprocess engineering and especially electrochemical bioprocess engineering. For example, cable bacteria could be used for electricity generation in microbial fuel cells (Li et al., 2020). By using CO_2_ as a carbon source, air is purified at the same time, which could be applied in exhaust gas purification, thereby contributing to the reduction of greenhouse gases. In the future, cable bacteria could also be used to convert CO_2_ into higher‐value products, for which, additionally, the product spectrum of cable bacteria needs to be analyzed in more detail. To enable the possible use of cable bacteria in these areas, suitable cultivation methods need to be developed in advance.

### 1.2 Development of synthetic sediment for cable bacteria growth

Cultivations described so far are mostly conducted in natural sediment, collected from the natural habitat of the respective cable bacteria strains (Bjerg et al., 2018; Li et al., 2022). However, Plum‐Jensen et al. were able to cultivate cable bacteria from one habitat on natural sediment from another habitat (Plum‐Jensen et al., 2024). Most cultivations are executed in simple reaction tubes with diameters between 4 and 5 cm, which are often integrated into liquid‐filled aquaria (fresh or salt water) with additional aeration (Marzocchi et al., 2022; Meysman et al., 2019; Risgaard‐Petersen et al., 2015; Schauer et al., 2014). This kind of setup makes it possible to study cable bacteria growth, metabolism, and influence on sediment. However, concerning the future applicability of cable bacteria in production processes, the use of natural sediments has several disadvantages. For once, natural sediment, even collected from the same water body, undergoes fluctuations. Parameters such as season, weather, or differences in the collection spot result in variations in sediment composition, for example, concerning concentrations of NH_4_^+^, NO_3_^−^, PO_4_^3−^, and SO_4_^2−^ (van de Velde et al., 2016). Because of this, reproducibility of results is restricted. Additionally, the exchange of locally extracted cable bacteria strains between research groups is complicated, due to difficulties in the long‐term provision of the respective sediment. Another possibility is the cultivation of obtained cable bacteria on locally available natural sediment, differing from the original habitat. However, this needs to be tested for each cable bacteria strain. When thinking of a possible scale‐up in the future, digging up natural sediment in larger quantities might have negative effects on the ecosystem and is thus not practicable. In conclusion, for microbial and molecular biological examination of cable bacteria, the investigation in the natural sediment is a good option; however, for possible utilization in productive bioprocesses or even in the industry, the use of a defined medium is necessary. Thus, the main objective of this study is the generation of synthetic sediment for the cultivation of cable bacteria. By this, growing cable bacteria in the lab is made easier and more reproducible, due to a defined and constant medium composition. Furthermore, this also opens up the possibility of a scale‐up for the future, thereby representing the first step toward the application of cable bacteria in bioprocess engineering.

## 2 MATERIALS AND METHODS

### 2.1 Preparation of natural sediment

Natural sediment was collected from Lake Gelterswoog (49.395031, 7.692260, Kaiserslautern, Germany) and was prepared based on the study by Thorup et al. (2021). Large components such as stones and leaves were removed by hand, then the sediment was mixed with a standard kitchen hand mixer. To remove the remaining bigger fragments, the sediment was sieved through a steel sieve with a pore size of 1 mm. The filtrate was then autoclaved for 45 min at 121°C. After cooling down, the sediment was homogenized by shaking and split into 40 mL portions into sterile 50 mL plastic reaction vessels (centrifuge tubes with base, Greiner Bio‐One). After this step, sediment was either directly used or stored at −20°C. Before cultivation, the sediment was centrifuged at 8000 *g* (centrifuge 383 K, Hermle Labortechnik GmbH) for 2 min, and the supernatant was transferred into new sterile 50 mL plastic reaction vessels. As much supernatant was transferred back into the sediment as was necessary to get a homogeneous, creamy consistency. To obtain anoxic sediment in the deeper sediment layers, the reaction vessels were placed for 30 min in a water bath (Memmert WNB22, Memmert GmbH + Co.KG) at 90°C for degassing. By this, the natural environment of the cable bacteria is mimicked, where filaments grow from the oxic surface sediment layer into the deeper, anoxic sediment. After cooling down, the sediment was then used for inoculation with cable bacteria.

### 2.2 Cultivation of *Electronema aureum* GS on natural sediment

For cultivation of the freshwater cable bacterium *Electronema aureum* GS (Thorup et al., 2021) on natural sediment, about 50 mg (wet weight) of an already growing culture was transferred with an inoculation pen to the surface of freshly prepared sediment. In this work, a starter culture of *E. aureum* was kindly supplied by Lars Peter Nielsen (Center for Electromicrobiology). The provided culture is a stable single strain culture of *E. aureum*, which, however, still contains other microorganisms than cable bacteria, occurring with *E. aureum* in its natural environment. This culture was then used to inoculate, respectively, prepared sediment from Lake Gelterswoog. Inoculated sediment was kept in a sterile cultivation box in an incubation cabinet at 22°C. The cultivation box was filled with ambient air. A thin layer of ~5 mm of supernatant over the sediment prevented the samples from drying out. If necessary, more supernatant was added during cultivation. After 2 weeks and always before further use of the cultures, sediment was checked for cable bacteria microscopically (Nikon Eclipse Ni; Nikon).

### 2.3 Analysis of soluble sediment components

Soluble sediment components were quantified with compact ion exchange chromatography (IC). A 930 Compact IC Flex (Metrohm AG) with an inline system for dialysis and a conductivity detector was used. Measurements of cations (Na^+^, NH_4_^+^, K^+^, Mg^2+^, and Ca^2+^) were conducted using a Metrosep C6‐250/4.0 column (Metrohm AG) at a flow rate of 0.9 mL min^−1^ of 4 mM nitric acid and 0.7 mM dipicolinic acid. Anions (Cl^−^, NO_3_^−^, PO_4_^3−^, and SO_4_^2−^) were measured using a Metrosep A Supp 5‐250/4.0 column (Metrohm AG) at a flow rate of 0.7 mL min^−1^ of 1 mM NaHCO_3_ and 3.2 mM Na_2_CO_3_. The oven temperature was at 35°C. Samples were prepared by centrifugation of the sediment for 15 min at 8000 *g* and subsequent filtration of the supernatant through a nylon filter (pore diameter 45 µm). For the measurement of cations, pH was adjusted to 3 ± 0.5 with 2 M HNO_3_. Measured ion concentrations were subsequently used for the design of the synthetic sediment.

### 2.4 Preparation of sand and bentonite for synthetic sediment

Different mixtures of sand and bentonite were used during the development of the synthetic sediment. Bentonite (CAS Nr. 1302‐78‐9) was ordered from Carl Roth GmbH and Co. KG. Three different sands were investigated: Sand was either (I) ordered quartz sea sand (particle size 205 µm (D50); CAS: 14808‐60‐7, VWR International GmbH) or quartz sand obtained from a local quarry (Carl Picard Natursteinwerk GmbH). Sand from the quarry was either only crushed with a mortar and sieved through a steel sieve (size 1 mm) or was additionally washed with 2 L deionized water per 100 g of sand (differentiation in the text from here on as (II) quarry sand and (III) washed quarry sand). Sand and bentonite were mixed in proportions of 70%–97.5% sand with 2.5%–30% bentonite (weight per weight).

### 2.5 Analysis of water retention of sand–bentonite mixtures

To evaluate the impact of proportions of sand–bentonite mixtures on water retention, mixtures of 100%, 97.5%, 95%, 90%, and 80% unwashed quarry sand with 0%, 2.5%, 5%, 10%, and 20% bentonite (w/w) were analyzed. Experiments were conducted with a total of 20 g solid weight and 10 mL of water in a 50 mL plastic reaction vessel. Sand–bentonite–water mixtures were placed in an incubation cabinet (BD 23; Binder GmbH) at 22°C analogous to the later cultivation experiment. The weight of the plastic reaction vessel was measured regularly for the determination of water evaporation. All experiments were carried out in triplicates.

### 2.6 Analysis of nickel content in different sands

Nickel content was examined for the quarry sand, the washed quarry sand, and the sea sand. Ten grams of sand with 20 mL deionized water in a 50 mL reaction tube were autoclaved and subsequently shaken at 80 rpm in a rotary shaker (IKA Trayster digital, IKA‐Werke GmbH & CO. KG) for 6 h. Afterward, nickel content was analyzed using a nickel trace cuvette test (LCK 537, Hach Company) with a range from 0.05 to 1.0 mg L^−1^ nickel. Measurements were conducted in an ultraviolet–visual photometer DR6000 (Hach Company).

### 2.7 Media components for synthetic sediment

Media components for the synthetic sediment were chosen based on the IC results of natural sediments and literature on cable bacteria metabolism. Additionally, the composition of the BG11 medium (Stanier et al., 1979) was used for orientation to make a selection of possible medium components and their concentrations. BG11 is a mineral medium for the cultivation of cyanobacteria, which also occur in most water bodies and are capable of autotrophic growth. Water soluble media components were NaNO_3_; K_2_HPO_4_ × 3 H_2_O, MgSO_4_ × 7 H_2_O, CaCl_2_ × 2 H_2_O; NH_4_HCO_3_, H_3_BO_3_, MnCl_2_ × 4 H_2_O, ZnSO_4_ × 7 H_2_O, Na_2_MoO_4_ × 2 H_2_O, CuSO_4_ × 5 H_2_O, and Co(NO_3_)_2_ × 6 H_2_O (see Table 1).

**TABLE 1:** Composition of medium and solid components for the developed synthetic sediment.

Medium components	Concentration (mg L^−1^)
NaNO_3_	150
K_2_HPO_4_ × 3 H_2_O	40
MgSO_4_ × 7 H_2_O	75
CaCl_2_ × 2 H_2_O	74
NH_4_HCO_3_	100
H_3_BO_3_	2.86
MnCl_2_ × 4 H_2_O	1.81
ZnSO_4_ × 7 H_2_O	0.22
Na_2_MoO_4_ × 2 H_2_O	0.39
CuSO_4_ × 5 H_2_O	0.08
Co(NO_3_)_2_ × 6 H_2_O	0.049
Demineralized water	

Solid components	Concentration (g L^−1^_medium_)
Sand + bentonite	2000
FeS	10

*Note*: Medium components were concentrated based on the composition of natural sediments and literature, solid components were based on literature and experiments on water retention and sample handling. The ratio of sand to bentonite is given in the corresponding experiments.

Components were dissolved in demineralized water and the pH was adjusted to 8 using NaOH or HCl. FeS (−100 mesh, 99.9% trace metals basis; CAS: 1317‐37‐9; Sigma‐Aldrich) was directly supplemented to the sand–bentonite mixture, due to its low solubility in water. Two grams of sand–bentonite mixture was mixed with 10 mL of medium and transferred to a 50 mL plastic reaction vessel. The next steps were conducted analogously to the treatment of the natural sediment: the synthetic sediment was autoclaved and subsequently placed for 30 min at 90°C in a water bath for degassing. After cooling down, the sediment was then used for inoculation with cable bacteria.

### 2.8 Cultivation of cable bacteria in synthetic sediment

For the cultivation of cable bacteria in synthetic sediment, 50 mg of preculture with sediment from Lake Gelterswoog was transferred to the surface of the prepared synthetic sediment. Three replicates were carried out for each cultivation. The inoculated sediment was placed in an incubation cabinet at 22°C and cultivated analogously to cultivations on natural sediment. Directly after inoculation and after 2 and 4 weeks, samples of 50–200 mg wet sediment were taken and microscopically analyzed.

### 2.9 Microscopic quantification of cable bacteria

To verify cable bacteria growth in the sediments, samples taken from cultivations were analyzed microscopically. Therefore, 50–200 mg of wet sediment was washed carefully in 300 µL of water to remove sediment particles. After the sediment had settled, 90 µL of the sample was transferred to a microscopic slide in three fractions of 30 µL covered with coverslips. The microscopic slide was placed under the microscope. All three fractions on the microscopic slide were completely screened under the microscope, cable bacteria filaments were counted, and pictures were taken for documentation. The values determined were then related to the amount of sediment used. To additionally determine the cable bacteria density in m cm^−3^, the length of the cable bacteria of later experiments was also evaluated using ImageJ (Schneider et al., 2012). For this purpose, all previously recorded images of the cable bacteria were analyzed using the “Segmented Line” tool. All fields of view containing cable bacteria were analyzed from two coverslips (1 × 1 cm) per replicate. Using the known scale of the images and the density of the sediment, the density of the cable bacteria could be determined in m cm^−3^. The density of the sediment was determined by measuring the volume of 1 g of sediment.

### 2.10 Setup of the sediment bioreactor

**FIGURE 1:**
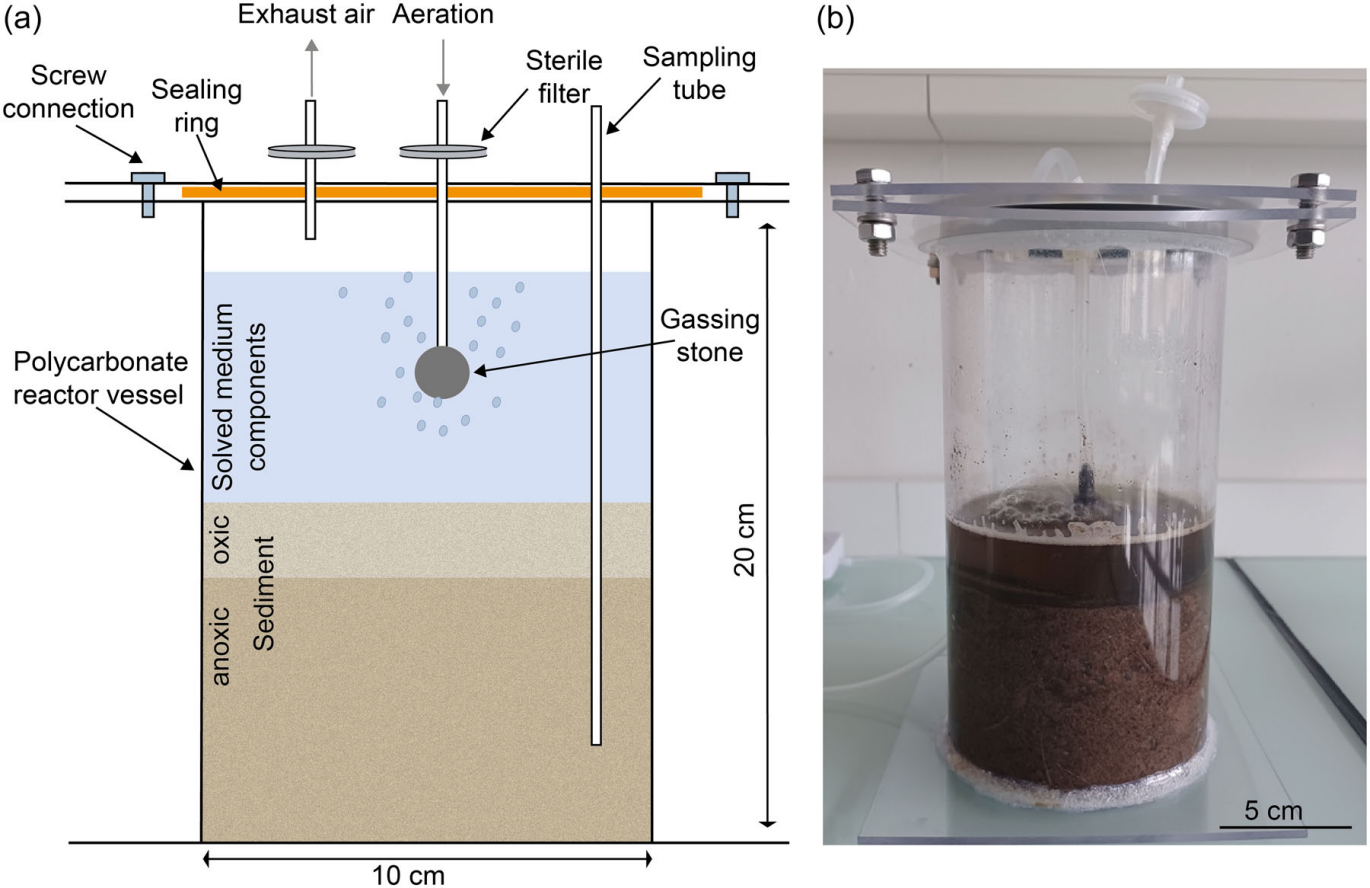
The developed sediment bioreactor for the cultivation of cable bacteria. (a) Schematic drawing of the reactor set‐up. The position of the sampling tube is only shown exemplarily. The sampling process by integration and removal of sampling tubes is possible at several positions. (b) Picture of the sediment bioreactor filled with sediment.

To be able to cultivate cable bacteria in a larger volume under more controlled conditions as in the reaction tubes used so far, a sediment bioreactor was developed (see Figure 1). A polycarbonate tube, bottom, and lid were ordered at KUS Kunststofftechnik. Polycarbonate was chosen since it can be autoclaved. The polycarbonate tube had a diameter of 110 mm, a wall strength of 3 mm, and a length of 200 mm. For the reactor bottom, a polycarbonate plate (150 × 150 × 3 mm) was used. For the construction of the lid, a combination of two polycarbonate plates was chosen: The first plate (150 × 150 × 3 mm) had a central hole with a diameter of 110 mm to fit around the polycarbonate tube. The second plate (150 × 150 × 3 mm) had five circular holes of ø 15 mm: one hole in the middle of the plate and four holes with a distance of 26 mm to the center of the plate. These openings were used in later experiments for integrating, for example, the gassing with air or sampling of the sediment. For this purpose, corresponding plugs were inserted with or without a feed‐through. The bottom plate, the polycarbonate tube, and the first plate of the lid were connected by gluing (UHU Allplast; UHU GmbH & Co KG). Between the two plates of the reactor lid, a sealing ring was placed and the plates were connected by four screws through drill holes (ø 6 mm). A gassing stone was integrated through the reactor lid and gassed with ambient air through a sterile filter. By aerating the medium above the sediment, an increased O_2_ concentration is obtained. By inserting a sampling tube, sediment can be taken to the desired depth and then analyzed layer by layer. This makes it possible to evaluate the growth of cable bacteria in different sediment layers. For cultivation in the sediment reactor, sediment was prepared analogously to cultivation in the 50 mL reaction vessel. Solid sediment components (sand, bentonite, and FeS) and soluble medium components were autoclaved, transferred to the reactor, and placed at 90°C for 30 min for degassing. Subsequently, the sediment was inoculated and examined microscopically as described before. Samples from three different positions in the reactor were analyzed.

## 3 RESULTS AND DISCUSSION

### 3.1 Cultivation of *E. aureum* on natural sediment from Lake Gelterswoog

**FIGURE 2:**
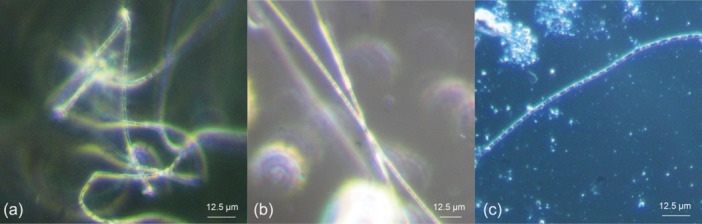
Microscopic pictures of *E. aureum* cultivated on natural sediment (a + b) and synthetic sediment (c). (a) Sediment from the original habitat (Aarhus, Denmark) and (b) sediment from Lake Gelterswoog after 25 days of cultivation. (c) Microscopic picture of first cultivation on synthetic sediment after 14 days of cultivation.

Since cable bacteria so far are only culturable in their natural sediment, as a first step, the cultivation of *E. aureum* was transferred to sediment obtained from the local freshwater lake Gelterswoog. This was done to maintain *E. aureum* on‐site in a growing culture and to generate biomass for inoculation of subsequent tests with synthetic sediments. 25 days after inoculation of autoclaved sediment from Lake Gelterswoog with *E. aureum*, cable bacteria filaments could successfully be detected microscopically (see Figure 2b), proving the applicability of the sediment for cultivation of *E. aureum*. When comparing microscopic pictures of *E. aureum* cultivated on sediment from Aarhus (Figure 2a) with sediment from Gelterswoog (Figure 2b), cable bacteria strains showed similar characteristics with a width of ~1.7 µm for both sediments. This also fits the published data for *E. aureum* with a cell width of 1.7 µm (Thorup et al., 2021).

In conclusion, *E. aureum* can be cultivated on natural sediment different from its original habitat. To assess whether this is a fundamental possibility for cultivating cable bacteria and thus could facilitate the worldwide exchange of cable bacteria strains between different locations, a similar approach would have to be tested with further cable bacteria strains and different natural sediments. A distinction should also be made between freshwater and saltwater cable bacteria, as different salinity requirements have already been described (Dam et al., 2021).

### 3.2 Test of varied sand–bentonite mixtures for the synthetic sediment

**FIGURE 3:**
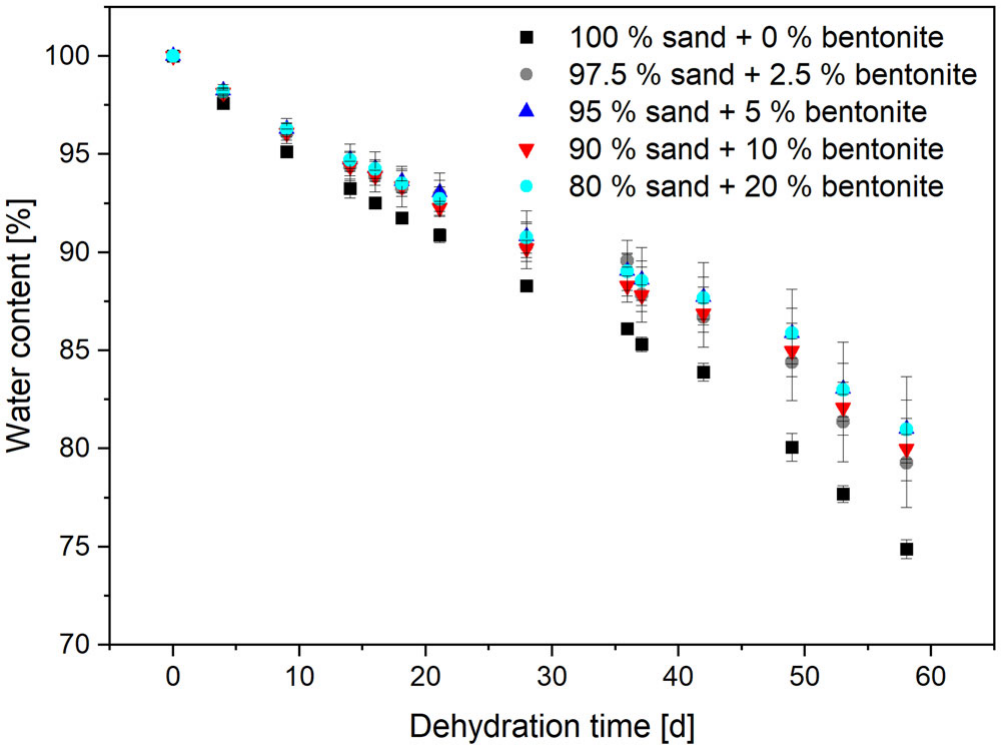
Influence of mixtures of different concentrations of sand and bentonite (w/w) on water retention. Experiments were conducted in 50 mL reaction tubes at 22°C in an incubation cabinet. Shown are means and standard deviations from *n* = 3 replicates.

A mixture of sand and bentonite was chosen for the solid components of the synthetic sediment. The water‐holding capacity of those mixtures was evaluated for possible cultivation of cable bacteria in the open air. Bentonite has a high water uptake capacity; however, too high bentonite concentrations result in a low permeability of the sediment (Proia et al., 2016). If the bentonite concentration is too low, the spaces between the sand grains are not completely filled and the water can evaporate more easily. Additionally, both materials are easily available in large quantities. The use of sand and clay (bentonite) was described before for synthetic sediments used in a different context: Le Bihanic et al. (2014) used a mixture of sand, clay, and organic matter for the cultivation of Japanese medaka larvae. They obtained the best results with a composition of 92.5% sand, 5% clay, and 2.5% organic matter. Marinkovic and Kraak cultivated *Chironomus riparius* larvae on synthetic sediment made of 75% sand, 20% clay, and 5% organic matter (Marinkovic & Kraak, 2010). Based on these results, the synthetic sediment mixtures of 80%–100% sand and 0%–20% bentonite (w/w) were tested. Higher bentonite concentrations resulted in a too‐compact sediment structure in preliminary results, which was already evident during the preparation of the sediment and was not analyzed in detail. With increasing bentonite content, water retention also increased (see Figure 3).

However, mixtures with high bentonite content were very compact and less comparable to the consistency of natural sediment than mixtures with less bentonite. Thus, the use of a lower proportion of bentonite (5% w/w) was chosen for further experiments. Since with 5% bentonite, after almost 60 days, still over 80% water remained, the amount of bentonite seemed still sufficient.

### 3.3 Composition of liquid medium components for the synthetic sediment

To obtain a first indication of the possible composition of the soluble components of the synthetic sediment, the nutrient compositions of supernatants from sediments from Aarhus and Gelterswoog were analyzed. Since *E. aureum* can grow in both sediments, compositions can give insight into necessary components and concentrations. Hereby, the sediment from Aarhus showed higher concentrations for all components but phosphate (see Table 2). However, since the growth of *E. aureum* in Gelterswoog sediment was also shown, concentrations seem to be sufficient. Possibly, in the long‐term cultivation, growth will cease earlier in sediment from Lake Gelterswoog due to limitations. Comparison with nutrient concentrations described in the literature shows variations in composition. Van de Velde et al. measured NH_4_^+^, NO_3_^−^, PO_4_^3−^, and SO_4_^2−^ in the overlying water from marine sediment of the coastal zone (van de Velde et al., 2016), with a maximum of 0.41 µM NH_4_^+^ (=7.54 µg L^−1^), 12.5 µM NO_3_^−^ (=0.74 mg L^−1^), 0.14 µM PO_4_^3−^ (13.30 µg L^−1^), and 27.3 mM SO_4_^2−^(=2.62 g L^−1^). Additionally, here a strong impact of season on concentrations was shown, which confirms the necessity of a synthetic sediment for reproducible cultivations.

**TABLE 2:** Composition of supernatant from sediment collected at Lake Gelterswoog in autumn (Kaiserslautern, Germany) and sediment from the natural habitat of *E. aureum* (Aarhus, Denmark) analyzed by IC analytic. Shown are single measurements. For comparison, the composition of the synthetic sediment is shown.

mg L^−1^	Na^+^	NH4^+^	K^+^	Mg^2+^	Ca^2+^	Cl^−^	NO_3−_	PO_4_^3−^	SO_4_^2−^
Gelterswoog	5.9	5.3	9.0	5.0	20.2	6.5	0.6	1.1	26.2
Aarhus	192.0	34.1	24.2	23.0	143.9	260.6	0.7	0.2	45.6
Synthetic sediment	40.61	22.82	13.71	7.40	20.17	36.34	109.45	16.65	29.33

In addition to IC analysis of sediment, literature was screened for already described substrates necessary for the growth of cable bacteria. Since only a few publications are available for *E. aureum*, data for other cable bacteria strains were also integrated. Cable bacteria have the ability for autotrophic CO_2_ fixation by dark carbon fixation via the Wood‐Ljungdahl pathway, allowing them to use inorganic carbon sources (Kjeldsen et al., 2019; Vasquez‐Cardenas et al., 2020). Additionally, hydrogen carbonate and propionate have been described as carbon sources for cable bacteria, however, based on their genome, *E. aureum* probably can only use hydrogen carbonate but not propionate (Kjeldsen et al., 2019). Thus, synthetic sediment was supplemented with ammonium hydrogen carbonate as a carbon source. Marzocchi et al. (2022) described the utilization of nitrate by *E. aureum*, whereby nitrate reduction to ammonium can be used as an alternative electron sink. Furthermore, the genetic potential for fixation of N_2_ has been shown for *E. aureum* (Kjeldsen et al., 2019). Since the importance of the availability of the different nitrogen sources for cable bacteria is unclear so far, an increased concentration of nitrate was added to the synthetic sediment. By aeration with ambient air, N_2_ and CO_2_ contained in the ambient air were added in subsequent cultures, but this should only affect the upper sediment layers. Since cable bacteria can produce phosphate granula as storage compounds (Geerlings et al., 2019; Kjeldsen et al., 2019), additionally an increased hydrogen phosphate content compared to natural sediment was added. Depletion of the electron donor has been described as one of the main reasons for dying off of cable bacteria communities (Seitaj et al., 2015). Accordingly, the addition of a sufficient amount of a suitable electron donor to the synthetic sediment is critical for the successful cultivation of cable bacteria. FeS has been described as a possible electron donor for cable bacteria in the anoxic sediment. Müller et al. could show a positive effect of adding additional FeS to natural sediment (Müller et al., 2016). Therefore, FeS was chosen as an electron donor in the synthetic sediment.

Based on the nutrient composition of the natural sediments, experiments on water retention, and findings from the literature, a synthetic sediment was designed (see Table 1). For better comparability with IC results, additional ionic concentrations of the synthetic sediment are given (see Table 2).

### 3.4 Cultivation of cable bacteria in synthetic sediment

**FIGURE 4:**
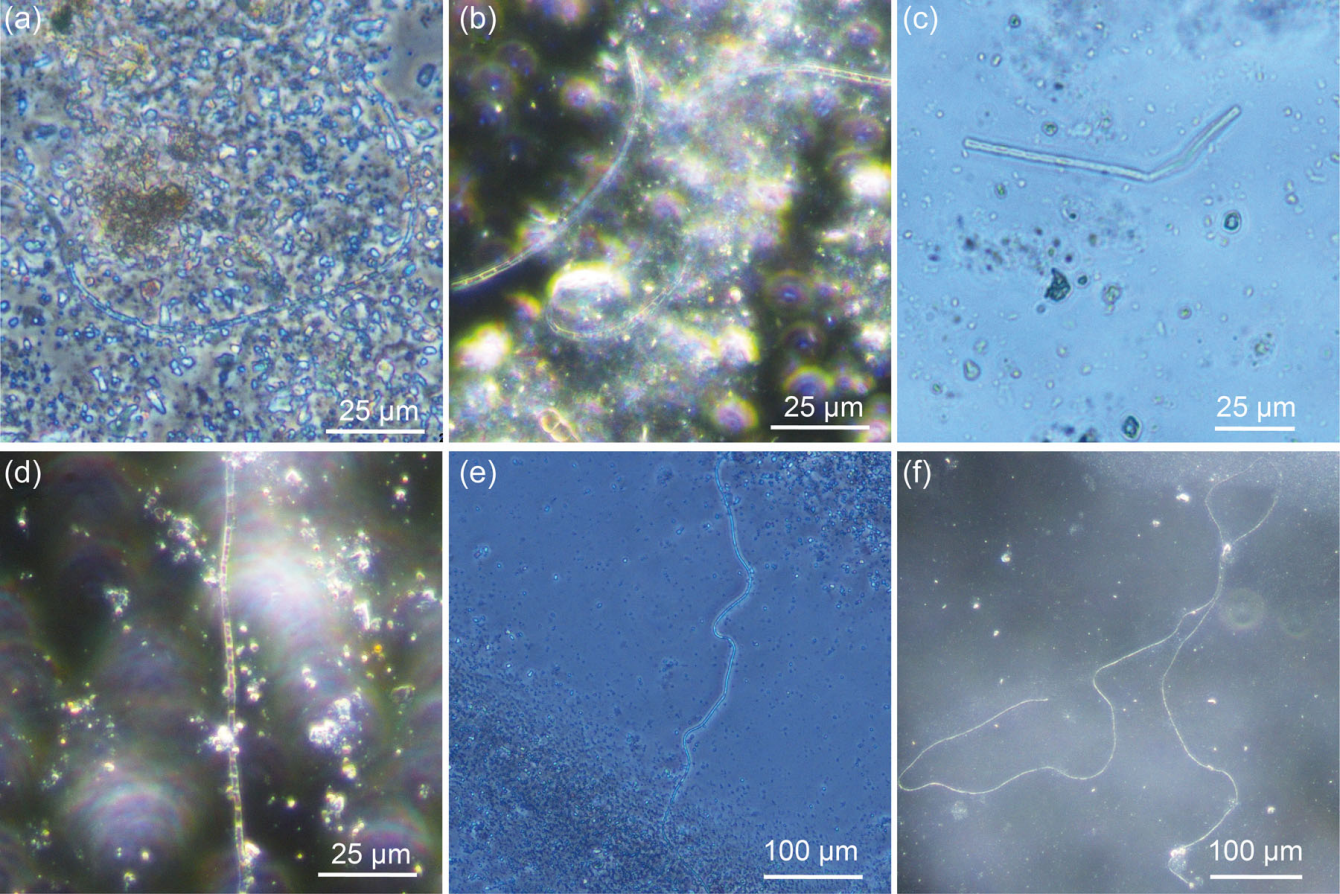
Microscopic pictures of *E. aureum* cultivated on synthetic sediment with quarry sand (a + d), washed quarry sand (b + e), or sea sand (c + f) after two (a–c) and four (d–f) weeks of cultivation. Cultivations were conducted in synthetic sediment in 50 mL reaction tubes at 22°C. Shown are exemplary pictures.

Cultivation of *E. aureum* in the synthetic sediment (95% sand + 5% bentonite) could be shown successfully 14 days after inoculation by microscopic analysis (see Figure 2c). However, small particles in the sediment made microscopic visualization difficult. To prevent this problem, in the next step different sources of sand were used and evaluated concerning its influence on cultivation and sample handling. Compared was sand from a sandstone quarry (=quarry sand), with the same sand with an additional washing step (=washed quarry sand) and sea sand. Furthermore, bentonite concentration was reduced to 2.5%. After 0, 2, and 4 weeks of cultivation, samples were taken and cable bacteria filaments were counted under the microscope (see Figure 4).

**FIGURE 5:**
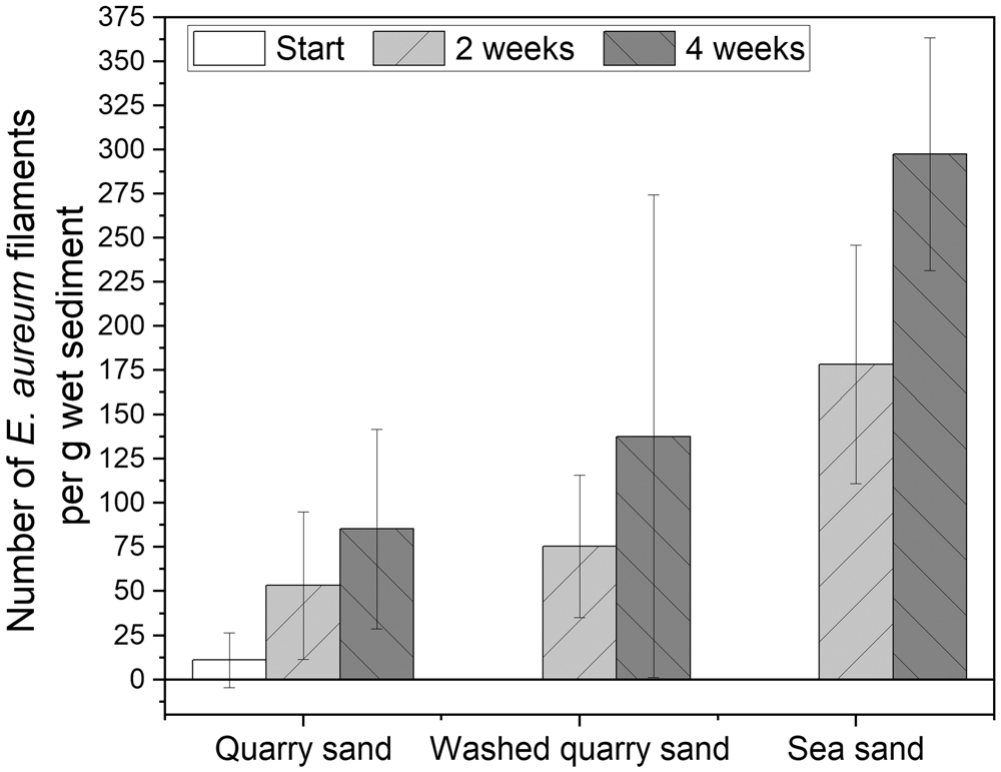
Number of *E. aureum* filaments in synthetic sediment with either sand from a sandstone quarry with and without washing step or sea sand. Cultivations were conducted in synthetic sediment in 50 mL reaction tubes at 22°C. After 0, 2, and 4 weeks wet sediment was harvested for microscopy. Shown are means from three replicates with standard deviations.

For all three cultivations, an increase in cable bacteria filaments could be shown after 2 weeks with a stronger increase after 4 weeks (see Figure 5). Thereby most cable bacteria were detected in sediment with sea sand, followed by washed sand from the sandstone quarry. In sand from the sandstone quarry without the washing step, the least number of cable bacteria could be seen under the microscope. This is not necessarily due to slower growth but small particles in the sand interfered with microscopic imaging (see Figure 4a), which could be circumvented by using the washed quarry sand. However, after 4 weeks, sea sand showed better results with 298 ± 66 compared to 138 ± 137 cable bacteria filaments per g sediment in washed quarry sand. Concluding, the sea sand showed the best characteristics concerning handling and microscopic imaging. Whether the sand also had an effect on growth, for example, because of varied pore size and easier penetrability for cable bacteria, still needs to be evaluated.

In literature doubling times of about 20 h are described for the first 10 days of a cable bacteria culture, in the following 10 days growth was slower and only another duplication was detected (Schauer et al., 2014). This fits the trends observed for the growth of *E. aureum* in the synthetic sediment, although the calculation of a doubling time for this method of cultivation is not possible. Since the synthetic sediment was only inoculated with a small number of cable bacteria and thus in the beginning, no cable bacteria filaments were detected in the samples, the calculation of a doubling time for the first 2 weeks of cultivation is not possible. Furthermore, only small amounts of sediment were taken and analyzed, and thus, not the complete number of cable bacteria filaments in the sediment was determined. Because cable bacteria can spread across the entire width of the sediment over time after inoculation due to their ability to migrate, evaluation of the entire sediment would be required to determine the actual growth rate. Boschker et al. describe the role of nickel in electron transfer over protein wires in the filament sheath (Boschker et al., 2021). Therefore, another important component of the sediment seems to be nickel. Since no nickel was added to the synthetic sediment but growth was still observed, it was analyzed, whether the sands already contained nickel. For the quarry sand, 25.5 mg_nickel_ kg_sand_^−1^ were determined for the washed quarry sand 9 mg_nickel_ kg_sand_^−1^ and the sea sand 23.5 mg_nickel_ kg_sand_^−1^. These values are in the range of nickel contents described for different sediments (Rinklebe & Shaheen, 2017) and therefore seem sufficient for cable bacteria growth. However, in future experiments, the impact of increased nickel contents should be evaluated.

These results show for the first time the successful cultivation of cable bacteria on synthetic sediment. Additionally, to the advantage of reproducibility of results, this could offer the possibility of generating pure cultures of cable bacteria. Until now, it has not been possible to fully isolate cable bacteria. Other microorganisms from the natural sediment community invariably remain in the culture. By transferring single cable bacteria filaments to the synthetic sediment and by consecutive inoculation steps of fresh sub‐cultivations on synthetic sediment, a generation of axenic cultures seems achievable, since the medium is less complex and thus more selective. For future cultivations, this would allow us to relate obtained results only to cable bacteria, and a possible effect of contaminants can be excluded. However, the success of this also depends on whether cable bacteria live in a necessary coexistence, which has not been proven so far. For example, the occurrence of diverse other bacteria was brought in connection with cable bacteria growth, however, it is not clear, whether cable bacteria also depend on the presence of these bacteria (Lustermans et al., 2023).

### 3.5 Cultivation in a sediment bioreactor prototype

**FIGURE 6:**
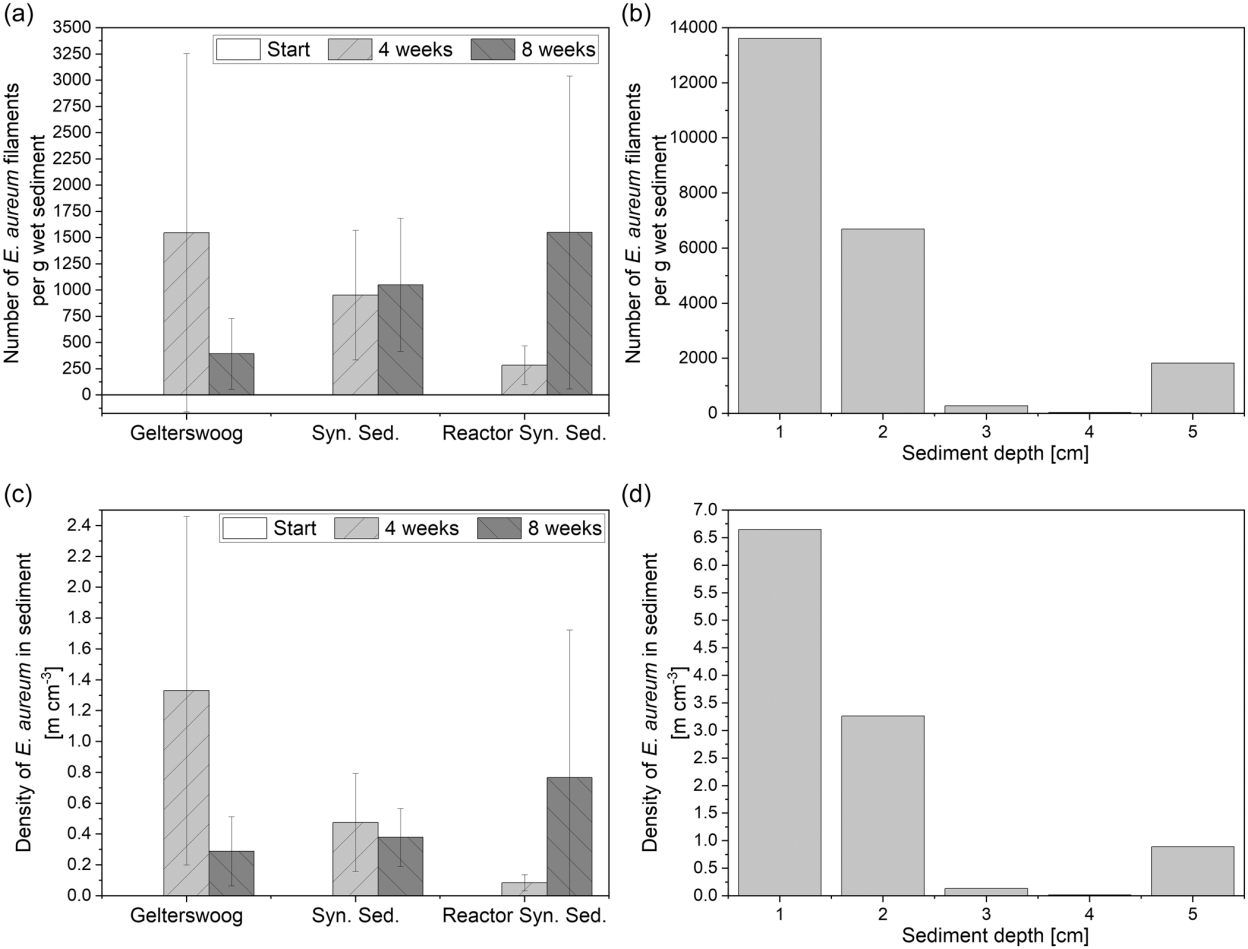
(a) Number of *E. aureum* filaments and (c) density of *E. aureum* filaments in sediment over a cultivation time of 8 weeks in 50 mL reaction tubes (RT) with either sediment from Lake Gelterswoog or synthetic sediment (Syn. Sed.) and in the developed sediment bioreactor with synthetic sediment. Cultivations were conducted at 22°C. Means and standard deviations for three replicates (reaction tubes) or from three sampling points (reactor) are shown. (b) Number of *E. aureum* filaments and (d) density of *E. aureum* filaments in sediment from the sediment surface up to a depth of 5 cm in the sediment bioreactor with synthetic sediment after a cultivation time of 10 weeks.

As a scale‐up of the experiment, *E. aureum* was cultivated in synthetic sediment in the developed sediment bioreactor for 10 weeks. The filament number and density of the cable bacteria in the sediment were examined and in addition, the sediment depth to which cable bacteria filaments could be found was investigated. Since the inoculation took place by bringing sediment to the surface, cable bacteria were initially only present in the upper sediment layers. To reach deeper layers, active migration of the cable bacteria and growth of the filaments was necessary to maintain a connection to the oxic sediment layer. For evaluation of growth in the sediment bioreactor, *E. aureum* was cultivated in the bioreactor in parallel with cultivations in 50 mL reaction tubes, both in synthetic sediment. Washed sand from the local quarry was used, due to its high availability and good results in previous experiments. This time, the bentonite was completely omitted from the synthetic sediment, since bentonite was added at the beginning mainly for water retention, and the risk of drying out in the reactor is reduced by a bigger layer of overlaying liquid. With the active aeration through the gassing stone, no limitation of oxygen in the overlaying liquid is expected, which allows the use of an increased liquid volume. Additionally, cultivations in 50 mL reaction tubes were conducted in natural sediment from Lake Gelterswoog, for comparison with the synthetic sediment. As before, in all cultivation set‐ups, an increasing number of cable bacteria filaments over time was observed (see Figure 6). Exemplary IC measurements of the pore water of two cultivations in the reactor also showed an increase in the sulfate concentration by 200% within 6 weeks. This may also be an indication of the oxidation processes caused by the cable bacteria (Burdorf et al., 2017). In the future, however, this should be investigated in more detail using microsensor measurements.

Compared to the previous experiment (see Figure 5), a higher number of cable bacteria filaments was observed in all cases. On the one hand, this could be due to the better microscopic analysis caused by the absence of bentonite. In addition, the vitality of the preculture could also have an influence, which should therefore be analyzed more closely in the future. After 2 weeks of cultivation with 1548 ± 1708 filaments per g wet sediment, the highest number of cable bacteria filaments was detected in sediment from Lake Gelterswoog in reaction tubes, compared to 953 ± 617 in synthetic sediment in reaction tubes and 285 ± 185 in synthetic sediment in the reactor. Considering the high standard deviation, the differences in the reaction vessels are negligible. The lower value in the reactor can be attributed to the fact that there is a much larger volume of sediment here, and therefore, a possible spread of the cable bacteria in the area results in a lower number of filaments at the sampling point. Cultivations on natural sediment show a decrease in cable bacteria filaments from four to 8 weeks. In comparison, higher filament densities were achieved after 8 weeks in synthetic sediment (50 mL reaction tubes with 1051 ± 634 and sediment bioreactor with 1549 ± 1491 filaments per g of wet sediment) than in natural sediment (393 ± 335 filaments per g of wet sediment). This indicates a possible limitation in the natural sediment due to the depletion of necessary compounds. This limitation probably does not occur in the synthetic sediment, since phosphate and nitrate were concentrated higher than the concentrations measured in the lake sediment (see Table 1). Another possible limiting factor could be the availability of the electron donor in the anoxic sediment layer, which has already been described as a reason for the death of cable bacteria (Seitaj et al., 2015). By the high availability of FeS as an electron donor in the synthetic sediment this limitation could be prevented. Additionally, after 10 weeks of cultivation, the distribution of cable bacteria from the sediment surface to a sediment depth of 5 cm was analyzed. Hereby, in 1 cm steps, the number of cable bacteria filaments was determined. The highest number of filaments was detected in the upper 1 cm of the sediment, followed by a depth of 2 cm. Even at a depth of 5 cm, 1825 cable bacteria filaments were found per g of wet sediment. This fits previous findings of cable bacteria in natural sediment in depths up to several cm (Risgaard‐Petersen et al., 2015). The low number of cable bacteria at 3 and 4 cm depth could be attributed to the way the samples were taken in relation to the filamentous growth of the cable bacteria. The samples were taken in one piece via a glass sampling tube and then divided into 1 cm thick sections. As the cable bacteria filaments extend from the anoxic sediment to the sediment surface, it is possible that the filaments in the middle sediment layers were pulled into either the upper or lower layers when the sediment areas were divided. Since the filament length may vary between counted single filaments, additionally, the filament length was measured. This allowed the calculation of density in m filament per cm^3^ sediment (see Figure 6c,d) and better comparability with literature values. The ratios of the densities in m per cm^3^ are similar to the ratios of the number of bacterial cable filaments counted (Figure 6a,b). After 10 weeks, a density of over 6 m cm^−3^ was reached in the top sediment layer. In comparison, in literature, densities from <2 up to >1000 m cm^−3^ are described for other cable bacteria strains (Marzocchi et al., 2018; Schauer et al., 2014; van de Velde et al., 2016). Further optimization of sediment composition and cultivation conditions are thus necessary. However, the aim of this study was not to achieve maximum values, but only to demonstrate the basic suitability of the synthetic sediment. Concluding, the obtained results show that cultivation in the developed synthetic sediment is possible and can also be successfully scaled up to the larger volume in the sediment bioreactor over a longer cultivation period.

## 4 CONCLUSION

In this work, for the first time, the design of a synthetic sediment for the cultivation of cable bacteria is presented. Hereby, information from the analysis of natural sediments was combined with literature data to set up a suitable composition for cable bacteria growth. Successful cultivation of the freshwater cable bacterium *E. aureum* could be proved microscopically after two and 4 weeks in 50 mL reaction tubes. By the development of a sediment bioreactor, a possible scale‐up was shown, with cultivations possible for at least 10 weeks. Although further optimization of sediment composition and cultivation setup is necessary, the use of synthetic sediment allows the cultivation of cable bacteria under defined conditions. However, additional measurements still need to be carried out in the future to confirm the activity of the cable bacteria, as so far, only microscopic counts and measurements of the cable bacteria filaments have been carried out. Microsensor measurements of pH, O_2_, and H_2_S in the sediment should be carried out to prove whether the profiles typical of cable bacteria also form in the synthetic sediment. In addition, the movement and, thus, the activity of the cable bacteria can be analyzed using trench slides. In addition, the migration of the cable bacteria in the sediment after inoculation should be analyzed in more detail. The cultivation of other freshwater cable bacteria strains on the synthetic sediment will be an interesting point for investigation as well as the adaptation of the synthetic sediment for marine cable bacteria strains.

In summary, the presented results represent a significant stride towards the potential application of cable bacteria in bioprocess engineering. A more detailed investigation into product formation could pave the way for the future utilization of cable bacteria in production processes. Their ability to fix CO_2_ could offer additional benefits. Moreover, due to their high conductivity, cable bacteria could be employed in electrochemical bioprocesses.

## AUTHOR CONTRIBUTIONS

**Judith Stiefelmaier**: Conceptualization (lead); funding acquisition (equal); Investigation (supporting); supervision (lead); writing—original draft (lead); writing—review & editing (lead). **Joshua Keller**: Investigation (equal); writing—review & editing (supporting). **Wiebke Neupert**: Investigation (equal); writing—review & editing (supporting). **Roland Ulber**: Conceptualization (supporting); funding acquisition (equal); Writing—review & editing (supporting).

## CONFLICT OF INTEREST STATEMENT

None declared.

## ETHICS STATEMENT

None required.

## Data Availability

All data generated or analyzed during this study are included in this published article.
